# Photoluminescent Spectral Broadening of Lead Halide Perovskite Nanocrystals Investigated by Emission Wavelength Dependent Lifetime

**DOI:** 10.3390/molecules25051151

**Published:** 2020-03-04

**Authors:** Jinlei Zhang, Jiuyang He, Lun Yang, Zhixing Gan

**Affiliations:** 1 Jiangsu Key Laboratory of Micro and Nano Heat Fluid Flow Technology and Energy Application, School of Mathematics and Physics, Suzhou University of Science and Technology, Suzhou 215009, China; zhangjinlei199119@126.com; 2 College of Physics and Electronic Engineering, Xinjiang Normal University, Urumqi 830054, China; hejiuyang@sina.com; 3 Institute for Advanced Materials, Hubei Key Laboratory of Pollutant Analysis & Reuse Technology, Hubei Normal University, Huangshi 435002, China; 4 Jiangsu Key Laboratory of Optoelectronic Technology, School of Physics and Technology, Nanjing Normal University, Nanjing 210023, China; 5 College of Materials Science and Engineering, Qingdao University of Science and Technology, Qingdao 266042, China

**Keywords:** perovskite nanocrystals, photoluminescence, bandwidth, lifetime

## Abstract

Despite intensive efforts, the fluorescence of perovskite nanocrystals (NCs) still suffers from a poor color purity, which limits the applications in light emitting and multicolor display. A deep understanding on the fundamental of the photoluminescent (PL) spectral broadening is thus of great significance. Herein, the PL decay curves of the CsPbCl_x_Br_3-x_ NCs are monitored at different wavelengths covering the entire PL band. Moreover, energy relaxation time τ and radiative recombination time β are obtained by numerical fittings. The dependences of τ and 1/β on the detection wavelength agree well with the steady-state PL spectrum, indicating the observed PL broadening is an intrinsic effect due to the resonance and off-resonance exciton radiative recombination processes. This work not only provides a new analysis method for time-resolved PL spectra of perovskites, but also gains a deep insight into the spectral broadening of the lead halide perovskite NCs.

## 1. Introduction

The lead halide perovskites nanocrystals (NCs) have attracted extensive interest due to their potential applications for low-cost and high-efficiency photovoltaic, light-emitting diodes (LEDs), and photo-detectors [1,2,3,4,5,6,7,8,9,10,11,12,13]. In particular, the mixed halide perovskite NCs, of which semiconductor bandgaps can be regulated by halide compositions and sizes, have attracted tremendous attention for flexible optoelectronic applications [14,15,16]. The optical response and emission wavelength can be tuned between ~ 1.5 and 3 eV, which enable the harvest of full solar spectrum and light emitting covering the entire visible range [1,2,3,4,5,6,7,8,9,10,11,12,13]. Thus, the colloidal perovskite NCs exhibiting high photoluminescence quantum yields (PLQYs) and the broad spectral tuneability have been widely reported [1,2]. For example, the all inorganic CsPbX_3_ (X = Cl, Br or I, or mixture) NCs with PL covering the entire visible spectral region of 410−700 nm were synthesized via compositional bandgap engineering and size effect [1].

However, the limited emission color purity due to the broad spectral width, have hindered the further development of high-performance perovskite LEDs [17,18,19]. Almeida et al. devised a general synthesis scheme for cesium lead bromide NCs which allows control over size, size distribution, shape, and phase (CsPbBr_3_ or Cs_4_PbBr_6_) by combining key insights on the acid−base interactions [19]. The PL full width at half maximum (FWHM) for CsPbBr_3_ nanocubes synthesized at different conditions are as large as 69–170 meV. Dong et al. developed a new synthesis approach to precisely control the size and improve the ensemble uniformity [17]. However, the FWHM of the ensemble PL is still as large as 94 meV for 6.2 nm NCs, 109 meV for 5.3 nm NCs, 117 meV for 4.1 nm NCs, and 142 meV for 3.7 nm NCs [17]. The challenge of producing fluorescent perovskite NCs with narrow bandwidth has been one of the major obstacles impeding the utilization of perovskite NCs. Such a broad emission band is commonly attributed to the size distribution in Cd- and Pb-based chalcogenide NCs [20,21]. Current efforts are focused on exploring of colloidal solutions of perovskite NCs with narrow size distributions to address this issue. Despite intensive efforts, these perovskite NCs are still suffering from a broad bandwidth. In fact, in addition to the size distribution, the PL spectral width could be attributed to complex factors, including intrinsic broadening, electron-phonon coupling, and inhomogeneous surface [22,23,24,25]. Moreover, the ion migration induced phase segregation also may lead to spectral broadening of mixed halide perovskite NCs [26,27,28,29]. Therefore, a fundamental exploration on the PL spectral broadening of the mixed-halide perovskite NCs is of great importance.

In this work, CsPbCl_1.32_Br_1.68_ NCs were synthesized by Protesescu’s method. The mixed halide perovskite NCs were selected to include the contribution of phase segregation. The colloidal CsPbCl_1.32_Br_1.68_ NCs solution shows excitation-independent emission centered at 465 nm. Transmission electron microscopy (TEM) shows the most probable size is 9.71 nm with a standard deviation of 2.94 nm. The PL decay curves of different emissions (450 to 485 nm) excited at the fixed wavelength (375 nm) are systematically measured. Also, energy relaxation time τ and radiative recombination time β are obtained by numerical fitting of the time-resolved PL decay curves. The β decreases firstly and then increases with the emission wavelengths and shows a maximum at emission of ca. 465 nm. The results indicate that the PL broadening is an intrinsic effect due to the resonance and off-resonance exciton radiative recombination processes.

## 2. Results and Discussion

**Figure 1:**
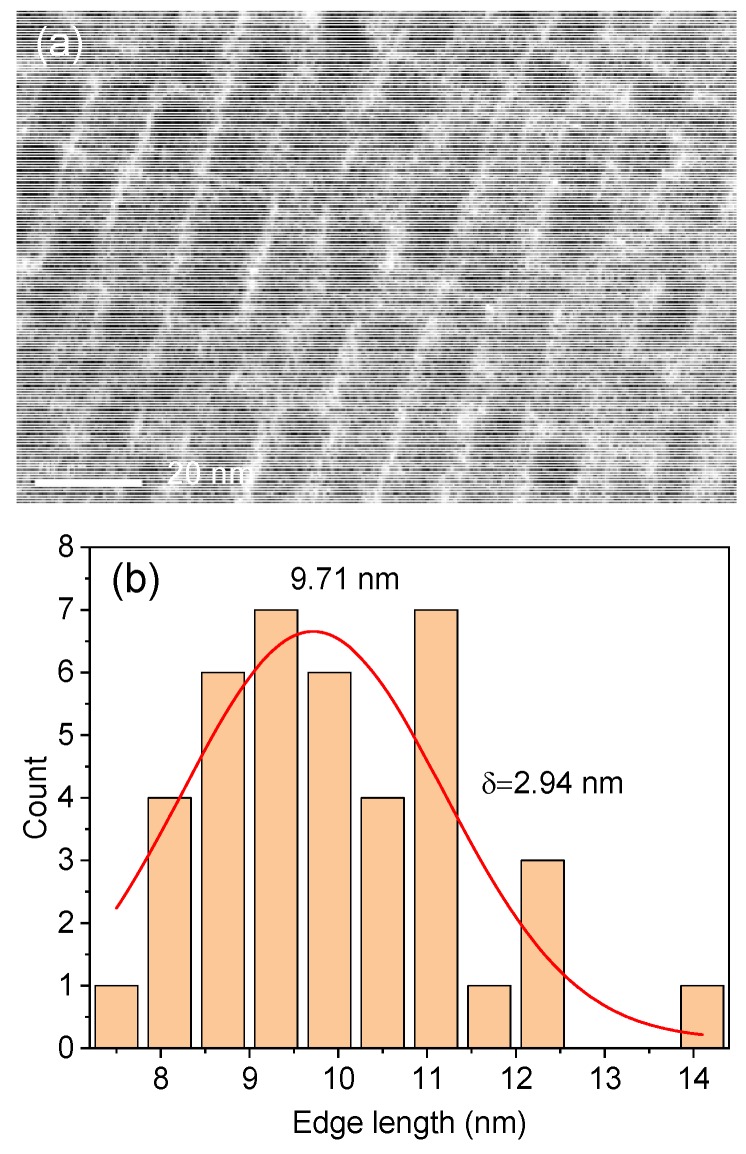
(**a**) TEM image of the perovskite nanocrystals (NCs) (**b**) Size distributions of the perovskite NCs calculated from the TEM image.

A representative TEM image of the perovskite NCs is shown in Figure 1a. The shapes of the perovskite NCs are cubic. The edge lengths’ distribution of the perovskite NCs calculated from the TEM image is plotted in Figure 1b, which is fitted by a Gaussian function. The most probable edge length of our perovskite NCs is 9.71 nm and the standard deviation is 2.94 nm. The uniformity of the perovskite NCs in this work is better than general preparation [30], while it is inferior to perovskite NCs with precise control, [19] representing a general situation. As is well known, size inhomogeneity is expected to be a main contribution to the PL broadening. Thus, in order to ensure the broad applicability of the conclusion and to include the possible contribution from size inhomogeneity, the synthesis in this work has not been optimized to eliminate the size in-homogeneity.

**Figure 2:**
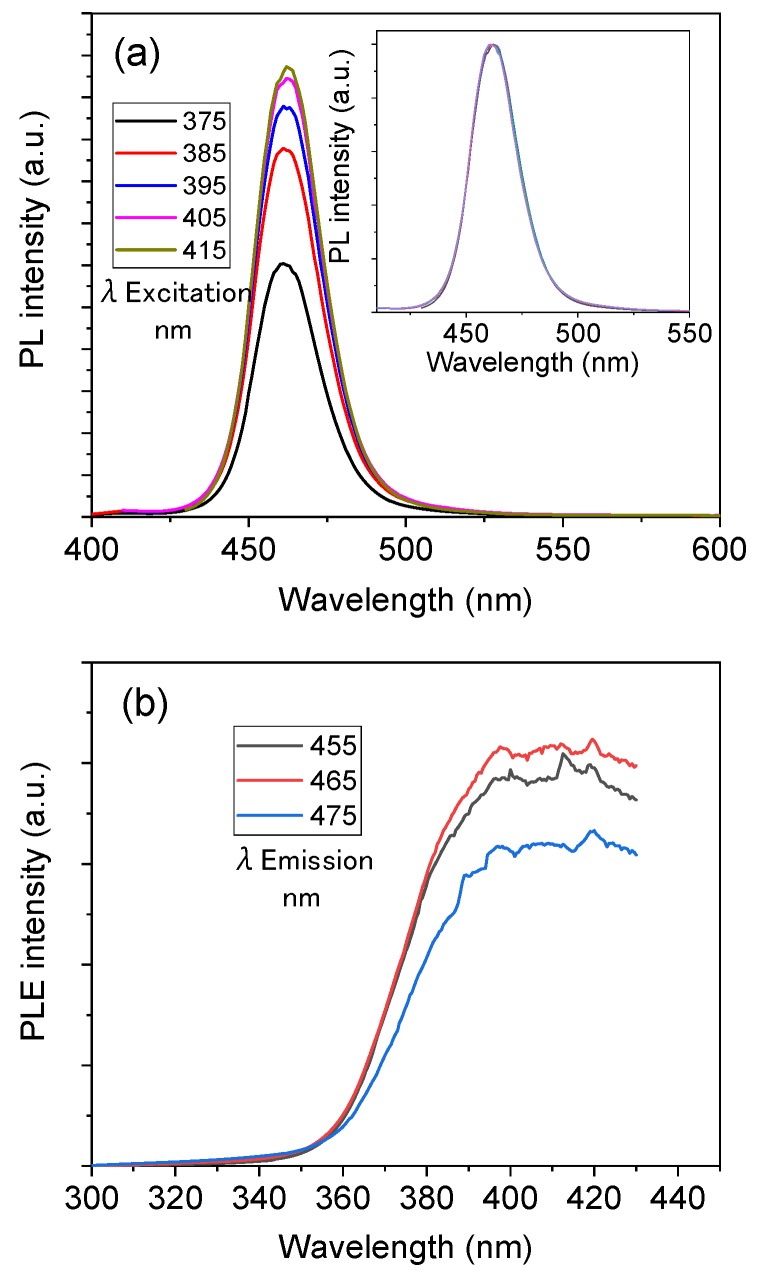
(**a**) Photoluminescent (PL) spectra excited at 375 to 415 nm with an interval of 10 nm. Inset: PL spectra excited at different wavelengths are normalized. (**b**) Excitation spectra monitored at emission wavelengths of 455, 465, and 475 nm.

PL spectra excited at 375 to 415 nm with an interval of 10 nm are shown in Figure 2a. The PL peak is ascribed to bandgap (2.65 eV) emission [31,32]. Except for different intensities, all the spectra almost keep the same peak position and spectral profiles. The FWHM is about 144 meV (25 nm), which is a reasonable value compared with other reports [17,18,19]. It is worth noting that redshift of PL spectra with increasing excitation wavelength is a fundamental feature of radiative recombination dominated by the size effect [31,32]. According to quantum confinement effect, the size distribution induces a corresponding distribution of the energy gaps. When excitation wavelength increases, more NCs with big sizes and narrow bandgaps are selectively excited, resulting in PL spectral redshift, which has been extensively observed in Si quantum dots, SiC NCs, carbon nanoparticles, graphene quantum dots, and MoS_2_ quantum dots [31,32,33]. Here, the PL peaks of perovskite NCs that hardly shift tend to imply minor contribution from size effect. PL excitation (PLE) spectra monitored at emission wavelengths of 455, 465, and 475 nm are also acquired. Similarly, the PLE spectra do not shift with the emission wavelengths. In addition, PL spectra excited at different wavelengths are normalized (Inset of Figure 2a). The PL spectral shape and FWHM almost keep the same when the excitation wavelength changes.

**Figure 3:**
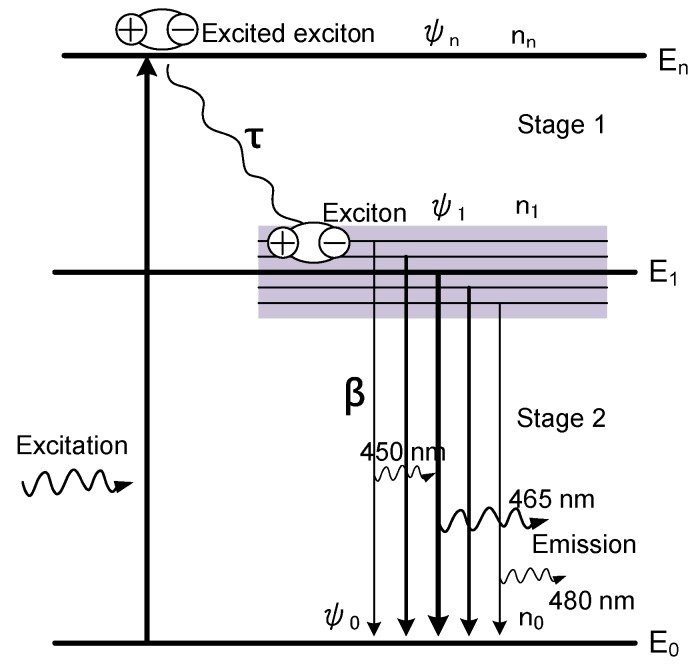
Schematic illustration of excitation, energy relaxation time τ and radiative recombination time β of an exciton in the perovskite NCs.

To explore the origins of the FWHM of colloidal perovskite NCs, we quantitatively study the time-resolved fluorescence decay spectrum of colloidal NCs as a function of the detection wavelength. The bandpass of the detector is fixed as small as 1 nm so that we collected only the fluorescence signals from NCs of one size, if NC size distribution is the sole cause of the broad FWHM. The emission wavelength dependent PL lifetimes are quantitatively analyzed by a model developed by Xu et al. [25]. This model was validated by both numerical simulations and experiments. In order to avoid repetition, this article does not go into too much detail. As schemed in Figure 3, the electron energy levels in a NC is characterized by quantized exciton levels. Upon photoexcitation, the photogenerated exciton is in an excited exciton state ψ_n_. The excited exciton relaxes to the ground exciton state ψ_1_ via nonradiative interactions, and then radiatively recombines to emit a photon. After that, the NC returns to its vacuum state ψ_0_. Before excitation, the NC is initially at its vacuum state. Once a pulsed excitation excites the QD at t = 0 so that n_n_ = 1 and n_1_ = n_0_ = 0. n_n_, n_1_, and n_0_ are populations of the state ψ_n_, ψ_1_, and ψ_0_, respectively. When ψ_n_-exciton nonradiatively relaxes to ψ_1_ at a rate of 1/τ while ground-state exciton radiatively transits to ψ_0_ at a rate of 1/β. The following rate equations can be acquired to describe the PL decay processes Equation (1):

(1) $$\{\begin{array}{c} \frac{dn_{n}}{dt}=-\frac{n_{n}\left(1-n_{1}\right)}{\tau }\\ \frac{dn_{1}}{dt}=\frac{n_{n}\left(1-n_{1}\right)}{\tau }-\frac{n_{1}\left(1-n_{0}\right)}{\beta }\\ \frac{dn_{0}}{dt}=\frac{n_{1}\left(1-n_{0}\right)}{\beta } \end{array}$$

Considering the Pauli exclusion principle, the transition efficiency of an exciton from an initial exciton level to a final exciton level is determined by both the occupation of the initial level and the un-occupation of the final level. For a very short time directly after the optical excitation, assuming ψ_1_, and ψ_0_ are empty, the PL intensity f_1_(t) is well characterized by a single decay term Equation (2):

(2) $$f_{1}\left(t\right)\propto e^{-t/\tau \prime }$$

The subscript “1” in $f_{1}\left(t\right)$ is assigned for the stage 1. τ′ depends on both τ and β. As decay proceeds, when n_n_ is negligibly small, the rate equations become Equation (3):

(3) $$\{\begin{array}{c} \frac{dn_{1}}{dt}=-\frac{n_{1}\left(1-n_{0}\right)}{\beta }\\ \frac{dn_{0}}{dt}=\frac{n_{1}\left(1-n_{0}\right)}{\beta } \end{array}$$

**Figure 4:**
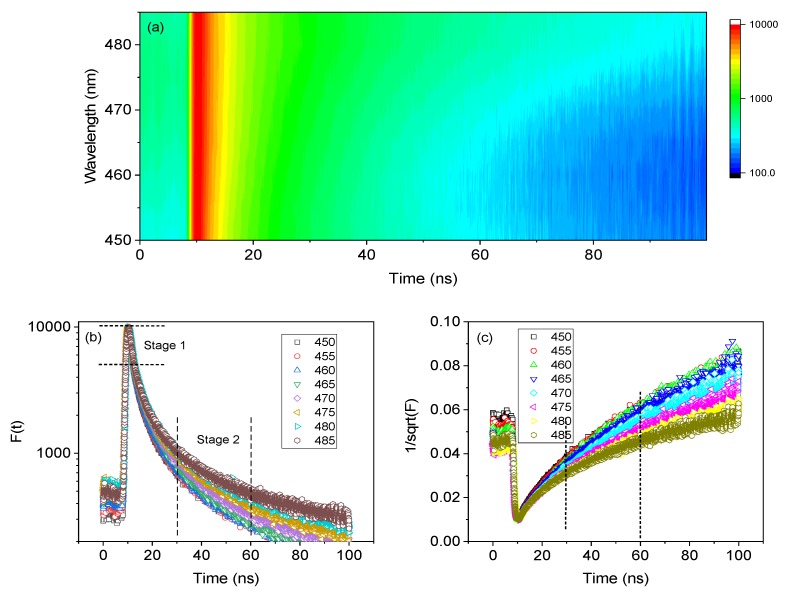
(**a**) The time-resolved PL decay mapping monitored at different detection wavelengths (450–485 nm) excited at a fixed wavelength (375 nm). (**b**) The time-resolved fluorescence decay spectra at different emissions (420, 460, 500, 540, and 600 nm). The detection bandpass was fixed to be 1 nm. Two stages are selected. (**c**) The time-resolved fluorescence decay curves are transformed to $1/\sqrt{F\left(t\right)}$ versus *t*.

The solution for the above equation is hardly exponential and needs many exponential decays to numerically fit it. However, at a special time stage $t\in \left(t_{1},t_{2}\right),$ $\left(1-n_{0}\right)=n_{1}=n_{0}^{\prime }$. The solution for above equation can be acquired Equation (4):

(4) $$\frac{1}{n_{1}}=\frac{1}{n_{0}^{\prime }}=\frac{t+a}{\beta }$$

where a is a constant. The long-time fluorescence decay, $f_{2}\left(t\right),$ at the second stage $t\in \left(t_{1},t_{2}\right)$ is Equation (5)

(5) $$f_{2}\left(t\right)=\int_{t_{1}}^{t_{2}}\frac{n_{1}\left(1-n_{0}\right)}{\beta }dt\propto \frac{\beta }{{\left(t+a\right)}^{2}}$$

since the variations of n_1_ and (1-n_0_) in *t* is very small in the time duration. The time-resolved PL decays of the perovskite NCs at different detection wavelengths (450–485 nm) excited at a fixed wavelength (375 nm) are obtained and shown in Figure 4. According to the model above, two different stages are defined in the PL decay curves (Figure 4b). The relaxation value τ is fitted by a single exponential function from the first stage of the PL decay function in the photon count range between 1 × 10^4^ and 0.5 × 10^4^. As shown in Figure 4c, the time-resolved PL decay curves are transformed to $1/\sqrt{F\left(t\right)}$, where F(t) is the fluorescence intensity as a function of decay time. The second-stage time window is found in the range of 30 and 60 ns, since $1/\sqrt{F\left(t\right)}$ is well approximated as linearly related to time t.

**Figure 5:**
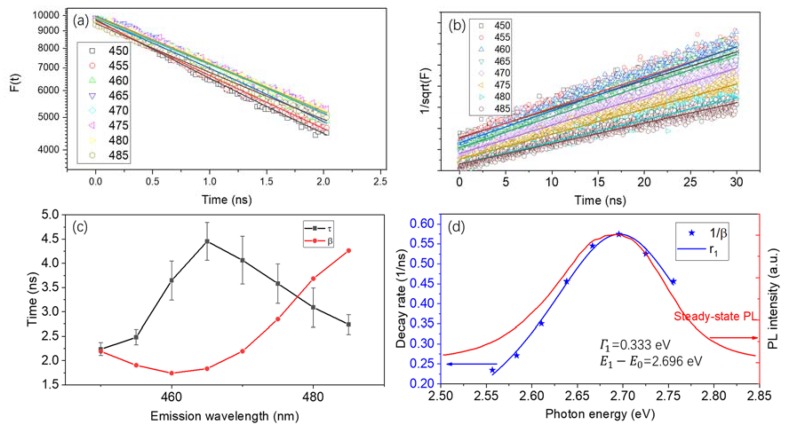
(**a**) The first stage of the PL decay curves fitted by simple exponential functions. (**b**) Linear fittings of $1/\sqrt{F\left(t\right)}$ versus *t* at the first stage area. (**c**) Fitting parameters as a function of detection wavelength. Black squares: τ; red circles: β. (**d**) The relationship between 1/β (blue star) and photon energy is fitted by Equation (6) (blue line). The steady sate spectrum (red line) is added for comparison.

The fittings of the two different stages are presented in Figure 5a,b, respectively. The acquired parameters as a function of detection wavelength are plotted in Figure 5c. The standard errors of fitting of *τ* are also presented. The results reveal that both τ and β depend strongly on the detection wavelength. β is minimal while τ is approximately maximum at the PL peak wavelength. The dependences of τ and 1/β on the detection wavelength agree well with the steady state PL spectrum. Very similar results have been observed for the conventional CdSe QDs [25]. According to the generalized Fermi golden rule, the decay rate $r_{1}\left(\hbar \omega \right)$ of exciton state ψ_1_ is given as [25]:

(6) $$r_{1}\left(\hbar \omega \right)=\frac{2\pi }{\hbar }{\left|\langle \psi_{0}|\sum_{i}V_{i}|\psi_{1}\rangle \right|}^{2}\frac{\Gamma_{1}}{\Gamma_{1}^{2}+{\left(E_{1}-E_{0}-\hbar \omega \right)}^{2}}$$

where ℏω is the photon energy, $V_{i}$ is the *i*th interaction between ψ_1_ and ψ_0_, and $\Gamma_{1}$ is the relaxation energy of ψ_1_ due to interactions $V_{i}$ with ψ_0_. According to Equation (6), resonance fluorescence with $E_{1}-E_{0}=\hbar \omega$ exhibits a shortest lifetime while off-resonance fluorescence ( $E_{1}-E_{0}\neq \hbar \omega$) possesses a long lifetime. As displayed in Figure 5d, Equation (6) is used to fit relationship between 1/β (blue star) and photon energy. The strong dependence of 1/β on the detection photon energy and their symmetry with respect to the emission wavelength indicate that the large FWHM of the perovskite NCs is most probably caused by intrinsic broadening rather than being dominated by the size distribution. Moreover, the steady-state spectrum (red line) is also added for comparison. The FWHM of the 1/β distribution peak is about 140 meV, which is very close to the FWHM of the steady-state PL spectrum (~144 meV). On the contrary, as reported previously, for PL of SiC NCs caused by size effect, both τ and β are hardly dependent on the detection wavelength [33]. In that case, the ultra-broad PL widths with FWHM>600 meV are originated from the size distribution of the NCs.

**Figure 6:**
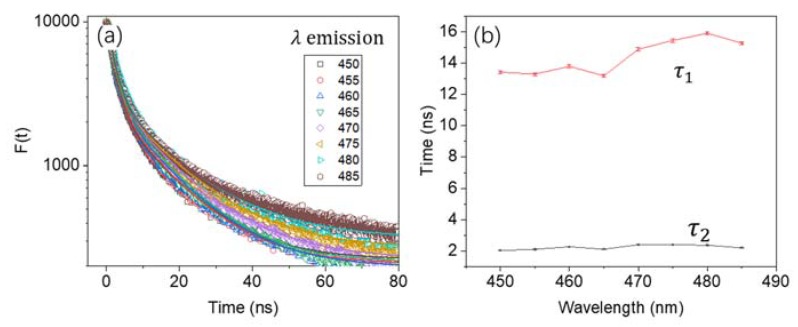
(**a**) Biexponential function fittings of the PL decay curves monitored at different wavelengths. (**b**) The two lifetimes $\tau_{1}$ and $\tau_{2}$ acquired by biexponential function fittings.

As found previously, the PL lifetime of perovskite NCs is strongly dependent on the halide composition, with Cl < Br < I. [1,34,35] The PL lifetime of the mixed halide CsPb(Br_x_Cl_1-x_)_3_ NCs should be in the range of 2.5 to 8 ns. In order to compare with previous reports, all the time-resolved PL results in this work are also simply fitted by conventional biexponential function: $F\left(t\right)=A_{1}\exp\left(-\frac{t}{\tau_{1}}\right)+A_{2}\exp\left(-\frac{t}{\tau_{2}}\right)$, with two lifetimes $\tau_{1}$ and $\tau_{2}$. Here, the PL lifetime ($\bar{\tau }=\frac{A_{1}\tau_{1}+A_{2}\tau_{2}}{A_{1}+A_{2}}$) of 465 nm emission is about 4.63 ns, indicating the perovskite NCs in this work are very common. As shown in Figure 6b, $\tau_{1}$ is about 13 to 16 ns while $\tau_{2}$ is about 2 ns. Except for a slight decrease of $\tau_{1}$ at the PL peak position (465 nm), the values of $\tau_{1}$ and $\tau_{2}$ do not exhibit notable dependence on the emission wavelength. In other words, the simple biexponential fitting is failed to investigate the origin of the PL broadening, since the $\tau_{1}$ and $\tau_{2}$ cannot be directly assigned to the energy relaxation and radiative recombination processes that are schemed in Figure 3.

## 3. Material and Methods

The colloidal CsPbX_3_ NCs solutions were synthesized by a method developed by Protesescu et al. [1]. Cs-oleate was prepared by dissolving 0.814 g Cs_2_CO_3_ into 100 mL 3-neck flask containing 40 mL octadecene (ODE) and 2.5 mL oleic acid (OA), dried at 120 °C for one hour, and then heated to 150 °C under protection of nitrogen gas until all Cs_2_CO_3_ reacted with OA. Then, 5 mL ODE and 0.188 mmol mixtures of PbBr_2_ and PbCl_2_ (0.052 g) were added into a 3-neck flask and dried under vacuum for 1 h at 120 °C. Dried oleylamine (0.5 mL, OLA) and dried OA (0.5 mL) were injected at 120 °C under N_2._ The temperature was raised to 140–200 °C, after complete solubilization of the PbX_2_. The Cs-oleate solution prepared earlier (0.4 mL,0.125M in ODE) was quickly injected. Five seconds later, the reaction mixture was cooled by the ice-water bath. Higher temperature of 150 °C and 1 mL of trioctylphosphine were required to solubilize PbCl_2_ for the synthesis of CsPbCl_3._ Finally, the aggregated NCs in the crude solution was separated by centrifuging. After centrifugation, the supernatant was discarded and stable solutions of colloidal CsPbX_3_ NCs were obtained by redispersing the particles in hexane. The concentration of the final CsPbX_3_ NCs solution is about 1 mg/mL. According to the Vegard’s law, [29] the chemical formula is estimated to be CsPbCl_1.32_Br_1.68_.

**Scheme 1:**
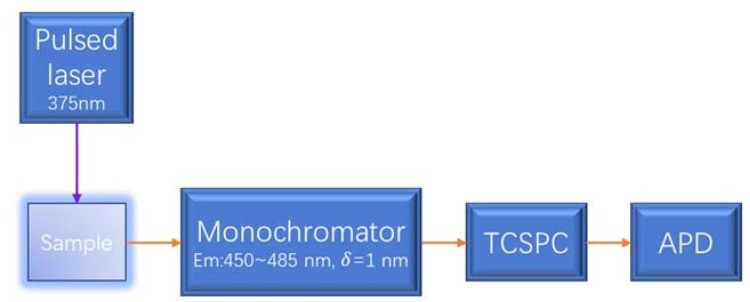
The optical set-up for measurements of time-resolved PL curves. TCSPC= time-correlated single photon counting system, APD= avalanche photodiode.

TEM images were captured by a JEOL JEM-2100F transmission electron microscope (TEM) (JEOL, Japan). The optical absorption spectra were obtained using a Shimadzu UV-2600 spectrometer (Shimadzu, Japan). Both steady-state and time-resolved PL (TRPL) measurements were performed on an Edinburgh FLS-920 fluorescence spectrometer ( Edinburgh Instruments, UK). As illustrated in Scheme 1, a 375 nm picosecond pulse laser was used as the excitation light (2 MHz, 0.5 mW) for TRPL measurements. The emission bandwidth was fixed at 1 nm by a monochromator equipped on FLS-920 spectrometer and the detection wavelength was modified from 450 to 485 nm to cover the fluorescence peak. Photon counting was stopped when the maximal number of photon counts reached a pre-set maximal value of 10^4^ in order to reach a high signal-to-noise ratio.

## 4. Conclusions

In summary, colloidal CsPbX_3_ perovskite NCs emitted at 465 nm with a bandwidth of 144 meV are synthesized. The all inorganic perovskite NCs solution shows excitation-independent emission and emission-independent excitation, implying a negligible contribution from inhomogeneous size. TEM image shows the size distribution of our perovskite NCs centering at 9.71 nm with a standard deviation of 2.94 nm. The PL decay curves of different emissions (from 450 to 485 nm) excited at the fixed wavelength (375 nm) are systematically measured. Moreover, energy relaxation time τ and radiative recombination time β are obtained by numerical fitting. The τ and β strongly depend on the detection wavelength. The β decreases firstly and then increases with the emission wavelengths, showing a maximum at PL peak position. The results further suggest that the FWHM of 144 meV of the perovskite NCs is most probably caused by the intrinsic broadening instead of inhomogeneous size distribution. We have further checked the CsPbBr_3_ NCs. Also, similar conclusion is also acquired. This work not only provides a new analyses of time-resolved fluorescence spectra of perovskites, but also gains a deep insight into the PL spectral broadening of the lead halide perovskite NCs. Based on this work, we now know that if FWHM is much larger than 144 meV, the broad PL and poor color purity may suffer from a poor size distribution. However, when the FWHM is even smaller than 144 meV, controlling on the uniformity of the NC size shows a limited ability to narrow the bandwidth.
